# Anatomical and Functional Results Following 23-Gauge Primary Pars Plana Vitrectomy for Rhegmatogenous Retinal Detachment: Superior versus Inferior Breaks

**DOI:** 10.1155/2017/2565249

**Published:** 2017-06-04

**Authors:** Panagiotis Stavrakas, Paris Tranos, Angeliki Androu, Paraskevi Xanthopoulou, Dimitrios Tsoukanas, Polixeni Stamatiou, Panagiotis Theodossiadis

**Affiliations:** ^1^2nd Department of Ophthalmology, University of Athens Medical School, Attikon University General Hospital, Athens, Greece; ^2^Ophthalmica Clinic, Thessaloniki, Greece; ^3^Ophthalmology Department, 401 Military Hospital, Athens, Greece

## Abstract

**Purpose:**

In this retrospective study, we evaluated the anatomical and functional outcomes of patients with rhegmatogenous retinal detachment primarily treated with pars plana vitrectomy in regard to the location of the breaks. Methods. 160 eyes were enrolled in this study, divided into two groups based on break location: the superior break group (115 eyes) and the inferior break group (45 eyes). The main endpoint of our study was the anatomical success at 3 months following surgery.

**Results:**

Primary retinal reattachment was achieved in 96.5% of patients in group A and in 93.3% in group B (no statistically significant difference, OR 1.98, 95% CI: 0.4, 7.7). Mean BCVA change and intraoperative complication rate were also not statistically significantly different between the two groups (*p* > 0.05, OR: 1.0, 95% CI: 0.9, 1.01, resp.). Statistical analyses showed that macula status, age, and preoperative BCVA had a significant effect on mean BCVA change (*p* = 0.0001, *p* = 0.005, and *p* = 0.001, resp.).

**Conclusion:**

This study supports that acceptable reattachment rates can be achieved using PPV for uncomplicated RRD irrespective of the breaks location and inferior breaks do not constitute an independent risk factor for worse anatomical or functional outcome.

## 1. Introduction

The primary use of pars plana vitrectomy (PPV) for the treatment of rhegmatogenous retinal detachment (RRD) has gained increasing popularity over the last few years as vitreoretinal surgeons become more familiar with this technique and recent technological advances contributed to improved anatomical and functional outcomes [1–14]. There are several advantages of PPV over scleral buckling (SB) technique including better visualization of the retina facilitating effective identification and treatment of all retinal breaks. In addition, adopting an internal approach makes it feasible to completely remove vitreoretinal traction and manage preretinal or subretinal proliferative vitreoretinopathy (PVR). Furthermore, the introduction of the new small-gauge transconjuctival sutureless systems is associated with less inflammation and patient discomfort as well as shorter recovery time [15–17].

However, RRD cases with inferior breaks may occasionally be challenging especially for phakic patients, in terms of completely removing the inferior vitreous and producing an effective endotamponading effect. In order to overcome the above shortcomings, a combined PPV and scleral buckling technique is often carried out to support the inferior retina, thus avoiding inconvenient posturing. However, this procedure is time-consuming and technically demanding, produces an increased risk of choroidal hemorrhage, and bears the risks of all the complications associated with SB, including refractive change, diplopia, explant erosion or infection, possible decreased retinal flow, and risk of anterior segment ischaemia [18–23].

During the last few years, an expanding published literature suggests that the adjuvant buckling procedure for RRDs with inferior breaks does not provide any statistically significant benefit as far as anatomical or functional success is concerned [24–28] and that the inferior location of the retinal break does not constitute a risk factor for anatomical failure [2, 29–31]. Additionally, it has been reported that small-gauge systems used for the repair of RRDs with inferior breaks are equally efficient to conventional 20 G techniques, despite the possible disadvantages of the increased flexibility of the instrumentation and the consequent insufficient peripheral vitreous removal and incomplete gas filling [32, 33]. On the other hand, there is a significant number of studies identifying the presence of an inferior break as a risk factor for anatomical failure [1, 11, 34–36] so that the initial surgical approach for RRD with inferior breaks remains a controversy.

The aim of our study is to compare the anatomical success rate and functional outcomes of patients with RRD primarily treated with PPV alone in regard to the location of the retinal breaks.

## 2. Materials and Methods

A retrospective computer- and intraoperative chart-based review of patients who were referred to the vitreoretinal service of the 2nd Department of Ophthalmology, University of Athens, was carried out. The institution's vitreoretinal database was used to select all consecutive patients who underwent vitrectomy for primary repair of RRD associated with superior or inferior breaks between January 2013 and March 2015. All consecutive patients with a RRD associated with superior or inferior breaks were included in the study.

Exclusion criteria were giant retinal tears, proliferative vitreoretinopathy (PVR) grade C or greater as defined by the Retina Society Terminology Committee [37], history of penetrating ocular trauma, retinal dialysis, previous ocular surgery excluding noncomplicated cataract extraction, and significant ocular comorbidities such as uveitis and glaucoma. Cases of concomitant presence of both superior and inferior breaks associated with RRD were also excluded.

Once suitable patients were identified, they were further subdivided into two groups. Group A consisted of patients with RRD associated with superior breaks, and group B consisted of patients with RRD associated with inferior breaks. A superior break was defined as a retinal break or hole present between 9 and 3 o'clock meridian whereas an inferior break was defined as a break or hole present between 4 and 8 o'clock meridian. Localization of breaks was ascertained on the basis of intraoperative findings instead of preoperative examinations, as indicated by a postsurgery report.

Preoperative evaluation consisted of a thorough examination including demographics, preoperative best-corrected visual acuity (BCVA), axial length, lens and macular status, and clinical features of retinal detachment including number and distribution of all retinal breaks and holes. Although preoperative retinal mapping is routinely performed for every case of retinal detachment for reasons of surgical planning, only data deriving from the postsurgery report were used for the purposes of this study, as mentioned above. Snellen VA was converted to the negative logarithm of minimal angle resolution (logMAR) for statistical analysis and comparison. Patient data are demonstrated in Table 1.

All patients underwent surgery by a single vitreoretinal surgeon (P.S.) under peribulbar anesthesia. A standard three-port 23 G pars plana vitrectomy was carried out using the Alcon Accurus (Alcon, Forth Worth, TX, USA) under a wide-angle viewing system (OCCULUS BIOM, Wetzlar, Germany). Phacoemulsification was only performed if the lens opacity was impairing the view during surgery. Core vitrectomy and peripheral shaving were carried out, and care was taken to relieve vitreous traction on all retinal breaks and suspicious degeneration areas. In all cases, cut rates ranged from 2200 to 2500 cpm to remove central vitreous and a fixed cut rate at 2500 cpm was used to remove peripheral vitreous. Following vitrectomy, an internal search was carried out with 360-degree indentation. All retinal breaks or holes as well as suspicious peripheral retinal degeneration areas were treated with either transscleral cryoretinopexy or endolaser depending on their position. A fluid/air exchange was subsequently performed via the most accessible retinal break. If there was no appropriate safe drainage site, a retinotomy was performed. Perfluorocarbon liquids were not routinely used as adjuncts to surgery. All patients underwent an air/gas exchange with either 20% sulfur hexafluoride (SF6) for group A or 14% octafluoropropane (C3F8) for group B as endotamponade agents. All patients were instructed to position their heads according to the localization of the breaks for only 24 hours postoperatively to encourage tamponade of retinal breaks and prompt absorption of subretinal fluid. No head positioning was advised to the patients after the first 24 hours postsurgery regardless of break location. No adjuvant SB procedure was used in either group. The follow-up varied from a minimum of 3 months to a maximum of one year.

The main endpoint of our study was the anatomical success as defined by complete retinal reattachment in the absence of any tamponade agent at 3 months following surgery. Functional outcome and intraoperative complications, namely, retinal incarceration, hemorrhage, crystalline lens touch, and inadvertent retina touch, were also recorded and analyzed. Further comparisons were made depending on lens status (superior phakic versus inferior phakic and superior pseudophakic versus inferior pseudophakic) as well as macula status (superior macula on versus inferior macula on and superior macula off versus inferior macula off). All combined cases were included in pseudophakic groups for statistical analysis, since the combined approach does not seem to influence the final anatomical result, which constitutes the main endpoint of our study [38].

Visual acuity measurements with Snellen charts were converted to a logarithm of the minimal angle of resolution (logMAR) for statistical analysis (counting fingers was converted to 1.40 logMAR and hand movements were converted to 2.70 logMAR [39]). We compared categorical variables using the chi-square test and continuous variables using the Mann–Whitney *U* test. Univariate and multivariable logistic regression analyses were performed to identify potential risk factors for anatomical restoration. Analyses were performed using SPSS v16.0 (SPSS Inc. Released 2007. SPSS for Windows, Version 16.0. Chicago, SPSS Inc.).

Institutional review board approval was obtained for this retrospective study by the local Ethics Committee. The research adhered to the tenets of the Declaration of Helsinki.

## 3. Results

One hundred and sixty eyes of 160 patients were included in this study. The mean age (SD) of the patients was 64.3 (11.7) years, and the male-to-female ratio was 3 : 2. Patients were divided into two groups according to the type of retinal detachment. Group A consisted of 115 eyes with retinal detachment caused by a superior break (superior break group), and group B consisted of 45 eyes with retinal detachment caused by an inferior break (inferior break group). The preoperative characteristics are shown in Table 1. The majority of the patients had a macula-off RD (89 out of 115 eyes in group A and 33 out of 45 eyes in group B). There were 79 phakic and 81 pseudophakic eyes in both groups. The mean number of breaks found intraoperatively was 1.62 ± 0.91 (range 1 to 4) for group A and 1.64 ± 0.93 (range 1 to 4) for group B. Only 5 patients from group A and 2 patients from group B received a combined phacoemulsification and intraocular lens procedure at the time of retinal detachment repair. There was no statistically significant difference between the 2 groups in terms of age, sex, preoperative visual acuity, macula status (on/off), intraocular lens status, and number of retinal breaks.

### 3.1. Primary Outcome Measures

Primary complete retinal reattachment following 23-gauge vitrectomy, retinopexy, and intraocular gas was achieved in 96.5% of patients in group A and in 93.3% of patients in group B. No statistically significant difference between the 2 groups was noted (Mann–Whitney *U* test; odds ratio 1.98, 95% CI: 0.4, 7.7).

Best-corrected visual acuity (BCVA) improved from 0.7 logMAR preoperatively to 0.4 postoperatively in group A and from 0.9 preoperatively to 0.3 postoperatively in group B. Comparing the mean change of preoperative and postoperative logMAR BCVA, there was no statistically significant difference between the two groups (Mann–Whitney *U* test, *p* > 0.05). Similarly, the patients in both groups presented no significant difference in an intraoperative complication rate (1.7% and 0.0% complication rates in group A and group B, resp.; odds ratio: 1.0, 95% CI: 0.9, 1.01).

### 3.2. Risk Factor Analysis for Anatomical Failure

Separate univariate analyses were performed to determine potential risk factors for anatomical failure (redetachment). The variables tested included the following: break location, age, sex, lens status (phakic/pseudophakic), intraoperative complications (yes/no), macular status before surgery (macula on/macula off), preoperative BCVA, and BCVA mean difference pre- and postsurgery. None of the variables was statistically significant (*p* value > 0.5). Univariate and multivariable logistic regression analyses were also performed to identify potential risk factors for retinal redetachment. However, due to the limited number of redetachments, the logistic regression models included only two predictors: break location and one of the following variables (age, sex, lens status (phakic/pseudophakic), intraoperative complications (yes/no), macular status before surgery (macula on/macula off), preoperative BCVA, and BCVA mean difference pre- and postsurgery), as well as the respective interaction term. There was no alteration in the statistical significance of the univariate analysis (Table 2).

### 3.3. Factors Affecting Mean Difference in BCVA Pre- and Postsurgery

Statistical analyses were performed using the Mann–Whitney *U* test to determine factors [anatomical restoration (yes/no), age, lens status (phakic/pseudophakic), intraoperative complications (yes/no), macular status before surgery (macula on/macula off), and BCVA before surgery] associated with mean change in BCVA following surgery. A 3-month period follow-up was used in order to reduce the possible bias caused by subsequent cataract development. Results showed that macula status, age, and preoperative BCVA had a significant effect on mean change in BCVA following PPV (*p* = 0.0001, *p* = 0.005, and *p* = 0.001, resp.). More precisely, younger patients with macula on retinal detachment and better presurgery BCVA had a more favorable outcome concerning mean change of BCVA following PPV (Table 3).

## 4. Discussion

This study constitutes, to our knowledge, one of the largest to date published series of uncomplicated rhegmatogenous retinal detachment treated with PPV and gas alone focusing on retinal break location. Results showed no significant difference in the final anatomical and functional outcomes between patients treated with vitrectomy for RRD with superior or inferior breaks. Similarly, break location did not affect intra- and postoperative complication rates. Interestingly, in our series, lens and macular status did not show any correlation with the anatomical outcomes, suggesting that the primary reattachment rate was independent on the extent of retinal detachment.

Up until recently, the presence of an inferior break would favor a combined PPV and SB procedure in order to support the inferior retina and thus increase the final success rate. However, the current study revealed a high reattachment rate with PPV alone, implying that undergoing a potentially more traumatic combined procedure might be unnecessary [5–14, 18–23].

At present, there is little published literature on the outcomes of vitrectomy and gas alone focusing exclusively on retinal break location. In addition, results of such studies are contradictory. In 1996, Heimann et al. [11] published a retrospective study of 53 patients studying the final outcome of PPV without adjuvant scleral buckle for RRD, stating that the final attachment rate for PPV alone for RRD associated with inferior breaks was 50%, which would clearly suggest a poor outcome for this group. However, the number of patients in the inferior break group was too small to draw any firm conclusions (6 patients). In 2009, Von Fricken et al. [34] studied the outcomes of vitrectomy for the repair of RRD using either 20 G or 25 G instrumentation in 125 eyes. They concluded that the success rate for inferior breaks was three times lower as opposed to that of superior breaks. Moreover, Abu El-Asram et al. [35] studied a consecutive series of 115 eyes with bullous RD treated with vitrectomy, concluding that the presence of inferior retinal breaks was significantly associated with a higher primary redetachment rate (*p* = 0.0156). However, perfluorocarbon liquids were routinely used during surgery, and encircling scleral bands were placed in all cases, rendering the procedure much more traumatic than PPV alone. In 2013, Goto et al. [36] compared the anatomical success rate of PPV alone for RRD with superior and inferior breaks of 82 eyes. The authors concluded that the primary anatomical success rate in the inferior group was significantly lower than that in the superior group (*p* = 0.012). However, only 20 of 82 eyes were associated with inferior breaks and 20% SF6 was used as an endotamponade agent in all cases irrespective of the localization of the breaks.

On the other hand, in 2011, Wickham et al. [29] produced a simplified formula to estimate the risk of failure and PVR following primary RRD repair by vitrectomy, where the localization of the breaks was not identified as a risk factor for anatomical failure. Sharma et al. [31] in a series of 48 eyes also concluded that there was no significant difference regarding anatomical and functional outcome between RRD associated with superior and inferior breaks treated with PPV alone. Similarly, Senn et al. [2], in 2002, while studying the role of PPV for pseudophakic RD, reported that the localization of the breaks had no influence on the anatomical or functional outcomes of 129 eyes included in their study.

In our study, we analyzed 160 consecutive eyes with a 96.5% success rate after primary vitrectomy in eyes with RRD associated with superior breaks and 93% success rate in eyes with RRD associated with inferior breaks and the difference in the reattachment rate proved to be nonsignificant. Our success rate is consistent with the high anatomical reattachment rates published in recent years in patients undergoing PPV for RRD [27, 30]. More importantly, our results further support the impression that inferior breaks do not represent a risk factor for retinal redetachment. Additionally, the phakic or pseudophakic status of the patient did not influence the final success rate either on the superior break group (group A) or on the inferior break group (group B). As far as the functional outcome is concerned, our analysis showed that macula status (macula on versus macula off), age, and baseline visual acuity were significantly related to the final BCVA.

The notion that PPV alone without scleral buckle may be enough in order to tamponade effectively the inferior retinal pathology seems to be increasingly adopted in published data over the last few years. Furthermore, acceptable rates of anatomical success using this method are being increasingly published: 89% for Wickham et al. [24] as well as for Tanner et al. [30], 81.82–92.68% for Dell'Omo et al. [32], 93.3% for Martinez-Castillo et al. [40], and 83.3–89.6% for Coyler et al. [33] and even in cases with PVR present (87.5% for Sheng et al. [41]) or even without postoperative head posturing (90% for Martinez-Castillo et al. [42]).

It is well known that retinal reattachment requires relief of traction, alteration of intravitreal currents, and chorioretinal adhesion [43]. A combined PPV-SB procedure does effectively address all three issues and, over the last few years, given the technological advances of PPV, the introduction of new small-gauge transconjuctival systems and panoramic viewing systems, and surgeons' growing confidence, so does PPV procedure alone. Relief of vitreous traction can be achieved with PPV alone for inferior retinal pathology, even for phakic patients, using new enhanced panoramic viewing systems in combination with scleral indentation bihanded approaches. This is also the case for addressing the issue of alteration of intraocular currents within the eye, since an almost complete vitreous clearance almost guarantees an adequate gas fill. The development of longer-acting gases and the elucidation of their physical properties have also contributed to PPV's high success rates, even for treating inferior retinal pathologies. Finally, chorioretinal adhesion is sufficiently addressed by the complete intraoperative subretinal fluid drainage in combination with retinopexy techniques.

There is a number of limitations in this study, including its retrospective nature as well as the lack of a control group of RRDs treated with combined procedure. However, already published data sufficiently address the issue of success and complication rates of the combined approach and since we have been applying our findings to our everyday practice over the last years, only a very limited number of combined procedures would have been available for statistical analysis. The uneven number of eyes with superior and inferior breaks is also a shortcoming; however, the consecutive nature of our data collection and analysis showed a prevalence of superior break RRDs compared to that of inferior break RRDs and any attempt to collect an equal number of eyes between the 2 groups would have generated bias to the statistical analysis.

We also excluded cases of concomitant superior and inferior breaks because the study set-up was to compare superior versus inferior breaks; thus, a third group of patients including both the main compared parameters would need a completely different study design.

The type of retinopexy used may also need clarification. We routinely use cryotherapy as retinopexy of choice in the presence of anterior breaks, regardless of their location. The advantages are simplicity of the procedure, since retinopexy can be applied even in the extreme periphery of phakic patients without a need for bimanual scleral indentation. In addition, cryotherapy can be applied in the presence of residual subretinal fluid as we avoid the routine use of perfluorocarbon liquids. However, in some of the cases of inferior breaks, endolaser was used instead of cryopexy. This was based on the assumption that endolaser creates chorioretinal scar in a shorter time compared to cryopexy; therefore, it would hold an advantage for breaks in the inferior quadrants that benefit from the endotamponade effects for a shorter postoperative period. We also used endolaser in cases of large inferior retinal breaks or breaks within areas of extended lattice degenerations that would otherwise require extended applications of cryopexy, thus causing a significant amount of inflammation.

In conclusion, this study provides further evidence that acceptable rates can be achieved using PPV alone to treat uncomplicated RRD irrespective of the location of the breaks. In this context, we suggest that additional SB procedure may be unnecessary even for eyes undergoing PPV for RRD caused by inferior breaks, since the latter does not constitute an independent risk factor for worse anatomical or functional outcome.

## Figures and Tables

**Table 1:** Distribution of preoperative variables in superior and inferior groups.

Characteristics	Superior break group	Inferior break group	*p* value
Age (y) [mean (SD)]	64 (±12)	66 (±12)	0.6^b^
Male/female (%)	74/40 (65/35)	33/9 (79/21)	0.12^a^
Presurgery visual acuity (logMAR)	1.2 (0.7)	1.37 (0.9)	0.8^b^
IOL status (yes/no) (%)	57/58 (50/50)	24/21 (53/47)	0.7^a^
Macula status (on/off) (%)	26/89 (23/77)	12/33 (27/73)	0.36^a^

^a^ *p* value obtained using Fisher's exact test; ^b^*p* value obtained using the Mann–Whitney–Wilcoxon test.

**Table 2:** 

Categorical variables		Restoration		
		Yes	No	*p* value
Sex	Male (*n* = 107)	102	5	0.6^a^
	Female (*n* = 49)	47	2	
Macula status	On (*n* = 38)	36	2	0.6^a^
	Off (*n* = 117)	111	5	
Intraoperative complications	Yes (*n* = 2)	2	0	0.7^a^
	No (*n* = 157)	151	7	
Phakic status	IOL (*n* = 81)	78	3	0.7^a^
	CL (*n* = 79)	75	4	
Break location	Superior (*n* = 113)	108	3	0.41^a^
	Inferior (*n* = 40)	37	3	

Categorical variables		Restoration		
		Yes	No	*p* value

Age		64 ± 11	67 ± 10	0.4^c^
Presurgery BCVA (logMAR)		1.2 ± 0.8	1.6 ± 0.95	0.1^b^

^a^ *p* value obtained using Fisher's exact test; ^b^*p* value obtained using the Mann–Whitney–Wilcoxon test; ^c^correlation is significant at the 0.05 level (2-tailed).

**Table 3:** 

Categorical variables		BCVA	
		Mean difference (SD)	*p* value
Macula status	On (*n* = 38)	0.25 ± 0.5	0.0001^a^
	Off (*n* = 117)	0.86 ± 0.65	
Intraoperative complications	Yes (*n* = 2)	0.6 ± 0.6	0.5^a^
	No (*n* = 157)	0.7 ± 0.7	
Phakic status	IOL (*n* = 81)	0.7 ± 0.7	0.3^a^
	CL (*n* = 79)	0.6 ± 0.66	
Break location	Superior(*n* = 113)	0.6 ± 0.5	0.2^a^
	Inferior (*n* = 40)	0.9 ± 0.9	
Restoration	Yes (*n* = 145)	0.68 ± 0.66	0.75^a^
	No (*n* = 8)	0.9 ± 1.0	

Categorical variables		BCVA	
		Mean difference (SD)	*p* value^b^

Age		0.273	0.005
Presurgery BCVA		0.85	<0.001

^a^ *p* value obtained using the Mann–Whitney–Wilcoxon; ^b^correlation is significant at the 0.05 level (2-tailed).

## References

[1] Heimann H., Zou X., Jandeck C. (2006). Primary vitrectomy for rhegmatogenous retinal detachment: an analysis of 512 cases. *Graefe's Archive for Clinical and Experimental Ophthalmology*.

[2] Senn P., Schmid M. K., Job O., Hürlimann A., Schipper I. (2002). Pars plana vitrectomy for pseudophakic retinal detachment. *Klinische Monatsblätter für Augenheilkunde*.

[3] Gerding H., Hersener A. (2013). Anatomical and functional results of primary pars plana vitrectomy in rhegmatogenous retinal detachment. *Klinische Monatsblätter für Augenheilkunde*.

[4] Mendrinos E., Dang-Burgener N. P., Stangos A. N., Sommerhalder J., Pournaras C. J. (2008). Primary vitrectomy for pseudophakic rhegmatogenous retinal detachment. *American Journal of Ophthalmology*.

[5] Miller D. M., Riemann C. D., Foster R. E., Petersen M. R. (2008). Primary repair of retinal detachment with 25-gauge pars plana vitrectomy. *Retina*.

[6] Hakin K. N., Lavin M. J., Leaver P. K. (1993). Primary vitrectomy for rhegmatogenous retinal detachment. *Graefe's Archive for Clinical and Experimental Ophthalmology*.

[7] Newmann D. K., Burton R. L. (1999). Primary vitrectomy for pseudophakic and aphakic retinal detachments. *Eye (London, England)*.

[8] Oshima Y., Emi K., Motokura M., Yamanishi S. (1999). Survey of surgical indications and results of primary pars plana vitrectomy for rhegmatogenous retinal detachments. *Japanese Journal of Ophthalmology*.

[9] Schneider E. W., Geraets R. L., Johnson M. W. (2012). Pars plana vitrectomy without adjuvant procedures for repair of primary rhegmatogenous retinal detachment. *Retina*.

[10] Figueroa M. S., López-Caballero C., Contreras I. (2010). Anatomical and functional outcomes for the treatment of pseudophakic rhegmatogenous retinal detachment. *Archivos de la Sociedad Española de Oftalmología*.

[11] Heimann H., Bornfeld N., Friedrichs W. (1996). Primary vitrectomy without sclera buckling for rhegmatogenous retinal detachment. *Graefe's Archive for Clinical and Experimental Ophthalmology*.

[12] Escoffery R. F., Olk R. J., Grand M. G., Boniuk I. (1985). Vitrectomy without scleral buckling for primary rhegmatogenous retinal detachment. *American Journal of Ophthalmology*.

[13] Campo R. V., Sipperley J. O., Sneed S. R. (1999). Pars plana vitrectomy without scleral buckle for pseudophakic retinal detachments. *Ophthalmology*.

[14] Gartry D. S., Chignell A. H., Franks W. A., Wong D. (1993). Pars plana vitrectomy for the treatment of rhegmatogenous retinal detachment uncomplicated by advanced proliferative vitreoretinopathy. *The British Journal of Ophthalmology*.

[15] Fujii G. Y., De Juan E., Humajun M. S. (2002). A new 25-gauge instrument system for transconjuctival sutureless vitrectomy surgery. *Ophthalmology*.

[16] Fujii G. Y., De Juan E., Humajun M. S. (2002). Initial experience using the transconjuctival sutureless vitrectomy system for vitreoretinal surgery. *Ophthalmology*.

[17] Kellner L., Wimpissinger B., Stolba U., Brannath W., Binder S. (2007). 25-gauge versus 20-gauge system for pars plana vitrectomy: a prospective randomized clinical trial. *The British Journal of Ophthalmology*.

[18] Flindall R. J., Norton E. W., Curtin V. T., Gass J. D. (1971). Reduction of extrusion and infection following episcleral silicone implants and cryopexy in retinal detachment surgery. *American Journal of Ophthalmology*.

[19] Hayashi H., Hayashi K., Nakao F., Hayashi F. (1997). Corneal shape changes after scleral buckling surgery. *Ophthalmology*.

[20] Domniz Y., Cahana M., Avni I. (2001). Corneal surface changes after pars plana vitrectomy and scleral buckling surgery. *Journal of Cataract and Refractive Surgery*.

[21] Fison P. N., Chignell A. H. (1987). Diplopia after retinal detachment surgery. *The British Journal of Ophthalmology*.

[22] Kwartz J., Charles S., McCormack P., Jackson A., Lavin M. (1994). Anterior segment ischaemia following segmental scleral buckling. *The British Journal of Ophthalmology*.

[23] Yoshida A., Feke G. T., Green G. J. (1983). Retinal circulatory changes after scleral buckling procedures. *American Journal of Ophthalmology*.

[24] Wickham L., Connor M., Aylward G. W. (2004). Vitrectomy and gas for inferior break retinal detachments: are the results comparable to vitrectomy, gas and scleral buckle?. *The British Journal of Ophthalmology*.

[25] Rush R. B., Simunovic M. P., Sheth S., Kratz A., Hunyor A. P. (2013). Pars plana vitrectomy versus combined pars plana vitrectomy-scleral buckle for secondary repair of retinal detachment. *Ophthalmic Surg Lasers Imaging. Retina.*.

[26] Weichel E. D., Martidis A., Fineman M. S. (2006). Pars plana vitrectomy versus combined pars plana vitrectomy-scleral buckle for primary repair of pseudophakic retinal detachment. *Ophthalmology*.

[27] Mehta S., Blinder K. J., Shah G. K., Grand M. G. (2011). Pars plana vitrectomy versus combined pars plana vitrectomy and scleral buckle for primary repair of rhegmatogenous retinal detachment. *Canadian Journal of Ophthalmology*.

[28] Kinori M., Moisseiev E., Shoshany N. (2011). Comparison of pars plana vitrectomy with and without scleral buckle for the repair of primary rhegmatogenous retinal detachment. *American Journal of Ophthalmology*.

[29] Wickham L., Ho-Yen G. O., Bunce C., Wong D., Charteris D. G. (2011). Surgical failure following primary retinal detachment by vitrectomy: risk factors and functional outcomes. *The British Journal of Ophthalmology*.

[30] Tanner V., Minihan M., Williamson T. (2001). Management of inferior retinal breaks during pars plana vitrectomy for retinal detachment. *The British Journal of Ophthalmology*.

[31] Sharma A., Grigoropoulos V., Williamson T. H. (2004). Management of primary rhegmatogenous retinal detachment with inferior breaks. *The British Journal of Ophthalmology*.

[32] Dell’Omo R., Barca F., Tan H. S., Bijl H. M., Lesnik Oberstein S. Y., Mura M. (2013). Pars plana vitrectomy for the repair of primary, inferior rhegmatogenous retinal detachment associated to inferior breaks. A comparison of a 25-gauge versus a 20-gauge system. *Graefe's Archive for Clinical and Experimental Ophthalmology*.

[33] Coyler M. H., Barazi M. K., von Fricken M. A. (2010). Retrospective comparison of 25-gauge transconjuctival sutureless vitrectomy to 20-gauge vitrectomy for the repair of pseudophakic primary inferior rhegmatogenous retinal detachment. *Retina*.

[34] Von Fricken M., Kunjukunju N., Weber C., Ko G. (2009). 25 gauge sutureless vitrectomy versus 20 gauge vitrectomy for the repair of primary rhegmatogenous retinal detachment. *Retina*.

[35] Abu El-Asram A. M., Al-Kwikbi H. F., Kangave D. (2009). Prognostic factors after primary pars plana vitrectomy and perfluorocarbon liquids for bullous rhegmatogenous retinal detachment. *European Journal of Ophthalmology*.

[36] Goto T., Nakagomi T., Ijima H. (2013). A comparison of the anatomic successes of primary vitrectomy for rhegmatogenous retinal detachment with superior and inferior breaks. *Acta Ophthalmologica*.

[37] Machemer R., Aaberg T. M., Freeman H. M., Irvine A. R., Lean J. S., Michels R. M. (1991). An updated classification of retinal detachment with proliferative vitreoretinopathy. *American Journal of Ophthalmology.*.

[38] Tosi G. M., Balestrazzi A., Baiocchi S. (2017). Previtrectomy phacoemulsification versus combined phacovitrectomy. *Retina*.

[39] Tan H. S., Mura M., Lesnik Oberstein S. Y., Bijl H. M. (2011). Safety of vitrectomy for floaters. *American Journal of Ophthalmology*.

[40] Martinez-Castillo V., Verdugo A., Boixadera A., Garcia-Arumi J., Corcostegui B. (2005). Management of inferior breaks in pseudophakic rhegmatogenous retinal detachment with pars plana vitrectomy and air. *Archives of Ophthalmology*.

[41] Sheng Y., Sun W., Mo B., Yu Y. J., Gu Y. S., Liu W. (2012). Non-buckled vitrectomy for retinal detachment with inferior breaks and proliferative vitreoretinophathy. *Int J Ophthalmol.*.

[42] Martinez-Castillo V., Boixadera A., Verdugo A., Garcia-Arumi J. (2005). Pars plana vitrectomy alone for the management of inferior breaks in pseudophakic retinal detachment without facedown position. *Opthalmology*.

[43] Machemer R. (1984). The importance of fluid absorption, traction, intraocular currents and chorioretinal scars in the therapy of rhegmatogenous retinal detachments. *American Journal of Ophthalmology*.

